# Is that really my movement?—Students' experiences of a video-supported interactive learning model for movement awareness

**DOI:** 10.3402/qhw.v10.28474

**Published:** 2015-08-12

**Authors:** Sofia Backåberg, Christina Gummesson, David Brunt, Mikael Rask

**Affiliations:** 1 Department of Health and Caring Sciences, Faculty of Health and Life Sciences, Linnaeus University, Växjö, Sweden; 2 Center for Teaching and Learning, Faculty of Medicine, Lund University, Lund, Sweden

**Keywords:** Video feedback, video modelling, reflection, phenomenological hermeneutics, observational movement analysis, nursing students

## Abstract

Healthcare staff and students have a great risk of developing musculoskeletal symptoms. One cause of this is heavy load related work activities such as manual handling, in which the quality of individual work technique may play a major role. Preventive interventions and well-defined educational strategies to support movement awareness and long-lasting movement changes need to be developed. The aim of the present study was to explore nursing students’ experiences of a newly developed interactive learning model for movement awareness. The learning model, which is based on a life-world perspective with focus on interpersonal interaction, has been used with 11 undergraduate students from the second and final year. Each student participated in three individual video sessions with a facilitator. Two individual interviews were carried out with each student during the learning process and one interview 12–18 months after the last session. The interviews were audio-recorded and transcribed verbatim, and a phenomenological hermeneutic method inspired by Paul Ricoeur and described by Lindseth and Norberg was used to interpret the interviews and diary notes. The interpretation resulted in three key themes and nine subthemes. The key themes were; “Obtaining better preconditions for bodily awareness,” “Experiencing changes in one's own movement,” and “Experiencing challenges in the learning process.” The interactive learning model entails a powerful and challenging experience that develops movement awareness. The experience of meaningfulness and usefulness emerges increasingly and alternates with a feeling of discomfort. The learning model may contribute to the body of knowledge of well-defined educational strategies in movement awareness and learning in, for example, preventive interventions and ergonomic education. It may also be valuable in other practical learning situations where movement awareness is required.

Musculoskeletal symptoms (MSS) have different origins and impact on all aspects of an individual's daily life. Healthcare professionals are one group at risk of developing MSS (Karahan, Abbasoglu, & Dogan, 2009), and among registered nurses, for example, the prevalence of long-lasting symptoms is 70–94% (Kyung Ja & Sung-Hyun, 2011; Smith et al., 2005; Smith, Wei, Kang, & Wang, 2004; Tinubu, Mbada, Oyeyemi, & Fabunmi, 2010). Physical load and manual work is one possible cause of MSS (Swedish Work Environment Authority, 2014), and an important risk factor for MSS is disadvantageous body posture during, for example, patient handling activities (Van den Heuvel, Ariëns, Boshuizen, Hoogendoorn, & Bongers, 2004). The everyday work situation for nurses includes a number of demanding tasks, such as manual handling activities, that is, patient transfers; work in challenging body positions; and computer work. MSS appear to begin during nursing education, and the prevalence of MSS during the previous 12 months has been shown to be 50–80% among nursing students (Backåberg, Rask, Brunt, & Gummesson, 2014; Kamwendo, 2000; Mitchell, O'Sullivan, Burnett, Straker, & Rudd, 2008; Smith & Leggat, 2004). A recent longitudinal study shows that neck/shoulder and back pain during nursing students’ final term remained constant for 2 years after finishing school, and one of the factors associated with the prevalence of symptoms was different types of physical load (Lövgren, Gustavsson, Melin, & Rudman, 2014). The individual performance, that is, the individual aspects of how the body is used in these demanding and repeated physical work situations, is another example of a factor that can influence the development of MSS (Kjellberg, Lagerström, & Hagberg, 2003). Thus, it is important to have access to long-lasting and effective interventions that prevent MSS among healthcare staff and undergraduate students.

The effect of ergonomic interventions among healthcare professionals has shown unconvincing results, and multifactorial interventions have thus been recommended in the literature (Jaromi, Nemeth, Kranicz, Laczko, & Betlehem, 2012; Thomas & Thomas, 2014). The focus of the interventions has not, however, been on educational aspects, which might be one explanation for the insufficiently beneficial results. How individual learning can be supported is an important aspect to gain sustainable change. In order to be able to change ineffective or compensatory movement patterns into physically gentle and effective ones, a certain amount of movement awareness is required (Everett & Kell, 2010). Awareness of one's own body has been described in two dimensions; how it is experienced, (i.e., experience dimension), and the actions and behaviour in movements and activities (i.e., movement dimension). These are aspects known to be important in rehabilitation, and a number of interventions for enhancing body awareness have shown beneficial results on health-related quality of life and cost-effectiveness (Gard, 2005; Gyllensten, Ekdahl, & Hansson, 2009). An intervention program in the ergonomic field with a focus on movement awareness and attitude changes connected to patient transfer situations has been evaluated with promising results on attitude changes over time (Kindblom-Rising, Wahlström, Nilsson-Wikmar, & Buer, 2011). However, it did not focus on changes in the individual movement pattern in everyday life.

Video feedback has been used in various ways within the healthcare context. In occupational health, video has been used to assess and evaluate posture and movements during work (Hakkarainen, Ketola, & Nevala, 2011; Soares et al., 2012). Within healthcare education and professional development, video feedback has shown to be beneficial in supporting postliminary reflection on in-action activities in group meetings (Carroll, Iedema, & Kerridge, 2008; Crenshaw, 2012; Iedema et al., 2009). Video feedback has also been considered to be useful in clinical skills training in nursing education as a basis for reflection about a training activity (Johannesson, Silén, Kvist, & Hult, 2013). Thus, video feedback has an important role in a number of learning situations, and it is often conducted subsequently as a single event and as a means for the learner's own further interpretation. To our knowledge, individual video-based feedback has seldom been used as an interactive tool to enhance movement awareness in everyday movements and not previously in an interactive model combined with reflection. Such a model has been described and studied by Backåberg, Rask, Gummesson, and Brunt (2015) with a focus on interpersonal interaction, showing that the model facilitates reflection and development of movement awareness and self-analysis. The results highlight the importance of a facilitator's role and flexible approach, for example, in promoting reflection. In the present study, the focus is on how the learning model is experienced by the participants, which has not been previously studied.

The main components of the learning model are video-based feedback, video modelling, and reflection. The ontological and epistemological theory of the lifeworld developed by Husserl (1859–1938) and further by Merleau-Ponty (1908–1961) has influenced the model, which means that the learner is being viewed as a subject with unique lived experiences (Ekebergh, 2007). The “lifeworld approach” has been described as the starting point for learning and understanding processes where reflection is essential (Ekebergh, 2007). In the model, this entails that openness and flexibility characterize the dialogue and that the process starts and follows the learner's reflection and learning process. The lifeworld perspective and its view of the learner is a foundation to which other contributory concepts have been added; observational learning theory (Bandura, 1986) and the system of building internal models (Elliott, Grierson, Hayes, & Lyons, 2011). The analysis of movement performance is based on the concept of observational movement analysis, in which the understanding of human movements as being initiated by force application against a surface is central (Everett & Kell, 2010; Hirschfeld, 2007).

MSS among healthcare staff are still a major problem and interventions to prevent symptoms need to be developed. Well-defined educational strategies to support long-lasting movement awareness and movement changes are lacking. The above-described learning model has shown potential to engage and motivate students to change their movement patterns and to develop movement awareness. However, we need more knowledge regarding experiences of using the model, and what it means over time to gain greater knowledge about the model and for its further development.

The aim of this study was to explore nursing students’ experiences of a newly developed interactive learning model for movement awareness.

## Method

### The learning model

The studied model is a video-supported interactive learning model for movement awareness described by Backåberg et al. (2015). The main components are video-based feedback, video modelling, and reflection, which are used in individual sessions with a facilitator. Self-selected video recordings for home practice and diary writing are also important parts of the model. In each session, preselected movements are performed and video recorded and the student has the opportunity to watch his/her own movement several times. The student's central role in the process and own reflection is emphasized and encouraged by open, reflective questions from the facilitator. One example of a probing question could be: What do you think of when you see your own movement? This was followed up by further questions regarding, for example, the student's experiences and feelings. Three sessions for each student were spread over a 4-month period.

### Setting and participants

The learning model was used with 11 nursing students and data for the present study were obtained from individual interviews and diary notes. Two individual interviews were carried out with each student during the learning process (prior to the second session and after the third session) and one follow-up interview of about 12–18 months after the last video session. The students were invited to write their reflections freely in a personal diary, how much and whenever they wanted, with a focus on experiences and thoughts regarding their own movements associated with the use of the learning model. At the end of the second and third year of study in a Bachelor of Science nursing program in Sweden, all nursing students were invited to participate in the study (105 students). They received written and verbal invitations and 21 students reported interest in participating. Five students dropped out prior to the beginning of the study and a further five dropped out during the study due to practical reasons, such as lack of time and travel difficulties, without providing any further explanation. A total of 11 students thus completed the sessions and interviews up to the follow-up interview, except for one, who only participated in two of the interviews, the first and the third, due to a period of placement abroad. The 32 interviews lasted between 20 and 52 min, and the first interviews were generally shorter than the follow-up interviews. The first and second interviews were carried out in a separate room at the clinical training centre, where the video sessions were completed. The follow-up interviews were either performed at the interviewer's office or via Skype if requested by the participant. The mean age of the 11 participants at baseline was 24.6 years (range 21–39) and 10 of them were women. Prior to the first session, the students completed a questionnaire concerning MSS and their impact on daily life physical activities. All 11 students reported MSS during the past 12 months and 5 of those reported impact on general physical activity. Nine students reported symptoms during the past 3 months (six of them reported impact on general physical activity).

### Data analysis

The experiences of the learning model were studied qualitatively with a phenomenological hermeneutic approach described by Lindseth and Norberg (2004), based on Ricoeur's philosophy of interpretation of a text (Ricoeur, 1976). Ricoeur maintains that in the attempt to decipher a text, there has to be a movement from the manifest content to the latent meaning of the text. He further means that understanding and explanation should not be understood in a dichotomy, but rather as being dialectically related to each other, to overlap and interact with each other in the interpretation process (Ricoeur, 1976).

The interpretation process is described by Lindseth and Norberg (2004) as consisting of three phases: the naïve reading, the structural analysis, and the comprehensive understanding. Data from all the interviews and diary notes were considered as a whole. In the naïve reading, all data were read several times with as much openness as possible and was then formulated as a naïve understanding. During the naïve reading and structural analysis, we tried to maintain a phenomenological approach, which entails changing from the natural attitude into being open, flexible, and reflective in order to grasp and describe the meaning of lived experiences. A thematic structural analysis was performed to grasp what the text talked about and to convey the essential meaning of lived experiences as condensed descriptions of each meaning unit. These were then reflected on regarding similarities and differences, which gradually led to the formulation of themes and subthemes. According to the method, the themes and subthemes were reflected on in relation to the naïve understanding to validate and adjust the naïve interpretation. In the comprehensive understanding, the themes and subthemes were reflected on in relation to the aim of the study and the interpretation, based on our pre-understanding, was carried out through critical reflection within the group of researchers. We also used relevant literature and philosophical theories to expand and broaden our understanding in relation to our findings. Ricoeur maintains that there is not only one probable interpretation of a text (Ricoeur, 1976; Ricoeur & Thompson, 1981), several interpretations were therefore tested and discussed within the group of researchers to find the most adequate interpretation corresponding to the research question.

### Ethical considerations

Ethical approval for the study was gained from the Regional Ethical Review Board in Linköping, Dnr: 2010/239-31. Verbal and written information about the study and the right to withdraw at any time without explanation were given to the students and they gave their written informed consent prior to the first session. None of the authors was involved as examiners to the participating students in their ordinary education and the facilitator in the sessions was not part of the research group.

## Results

### Naïve understanding

The students describe the learning model as providing greater knowledge and understanding of their own movements. To watch oneself on the film seems to initially imply an unpleasant experience, which is at the same time instructive and useful. The role of the facilitator is experienced as important for creating a comfortable and permitting atmosphere. The facilitator's reflective inquiries appear to provide a basis for self-reflection and support the student's learning. Being able to initially describe one's own movements in words, and when needed receive guidance from the facilitator, is seen as being supportive for movement changes. The learning may be experienced as difficult as the changed movements feel unaccustomed for the students, and a focus on details in the analysis may confuse the students when performing the movements. New ideas appear to emerge within the students, not only during the performance of the movements but also in everyday life situations and interactions with others.

### Structural analysis

The structural analysis resulted in three key themes and nine subthemes illustrating nursing students’ experiences of the learning model. The subthemes are described under each theme followed by the comprehensive understanding. An overview of themes and subthemes is presented in Table I.

**Table I:** Overview of key themes and subthemes.

Obtaining better preconditions for bodily awareness	Experiencing changes in one's own movement	Experiencing challenges in the learning process
Experiencing awareness by encountering one's own movement Using one's new awareness Experiencing cognitive impact for change Experiencing change as meaningful Being supported in self-reflection	Experiencing a changed movement Changing the image of one's own movement	Experiencing uncertainty Experiencing difficulties when changing

#### Obtaining better preconditions for bodily awareness

The students’ awareness of how they moved and used their body in everyday life increased when they used the learning model, which was experienced as meaningful. The interaction with the facilitator was essential for supporting, encouraging, and guiding the students during the learning process, and the attitude of the facilitator was emphasized as important. Having a pleasant and permitting atmosphere in the room was important for being able to feel comfortable in the situation. Watching oneself awkwardly performing the ordinary movements generated feelings of concern. Being aware of the possibilities for influencing future load-related symptoms was, however, experienced as motivating for long-lasting change.

##### Experiencing awareness by encountering one's own movement

In the beginning, the students felt uncomfortable being in the video-recording situation and watching themselves, but at the same time they found it both useful and instructive. The facilitator's uncritical, non-judgmental attitude was experienced as important, being as the situation, from the beginning, may have been experienced as uncomfortable and vulnerable.

I think that it's awful being on film and needing to see oneself … it's the worst that can happen … but gradually I began to think that it was very good … and while it was going on it was very good. (student no. 6).

Watching one's own movement on the film was important for the students in becoming aware of their own movements. Comparing one's own films with the films of the role model, as well as being able to see the films in slow motion, was important for the students to discover details and to expand their understanding of their own movements. That the learning process starts with one's own films was experienced as more supportive for learning in comparison with just watching someone else demonstrating how the movement should be performed. The individual perspective was seen as generating a greater focus and awareness of one's own movement pattern and the students thought it was important to identify shortcomings on their own.

Hmm … it was just seeing myself and being able to see what was wrong and compare … then I could think a little about it and I think it's important … yes … that was the best … when you are allowed to think yourself and try to see what you've done wrong … that's it I think. (student no. 8)

Breaking down the material and focusing on elements of the movement in relation to the whole movement were experienced as positive and important for learning. It was important for the students to be able to both see and feel the changes as well as being able to repeat the movements and to try various ways of performing them. The possibility of repeating the movements several times provided the students with confidence and allowed them to feel better at performing them.

Yes it was good to get feedback … and to see what I had done wrong or what should I call it … hmm … and that I could practice it and that I could do it twice more, then it was also really good as I got the chance to … hmm … correct it and then I didn't feel so bad (laughter). (student no. 3)

It was experienced as facilitating and important to have some prior knowledge about the prerequisites for how to perform natural body movements and gentle work techniques. Some students felt that they would have liked to have more movements to practice whereas others did not. Some were surprised about how many changes in posture and movement patterns were made, despite the relatively short time for training, this was something they had not expected.

Being as I didn't think that one could change like that … it hasn't taken a long time, it's been about three sessions and you don't think you'll be able to use anything at all and I don't think I spent much time on it either … especially … but something seems to have stuck I think … you're always surprised when you check and suddenly I've changed it … yes it was interesting so … I think it's been positive … great fun actually to see how one does it and how one can change so quickly … hmm … it's felt good. (student no. 9)

##### Using one's new awareness

The students felt that they had become more aware of their body movements in everyday situations. They had begun to reflect more upon their own movements both at home and at work although it was experienced as being easier to be aware at work.

Walked in the woods with the dog and noticed very clearly that I didn't stumble as much when I was careful about putting weight on my foot on the little toe side and then push off with my big toe. Greater stability in my knee I think? (student no. 1, diary)

The students felt that they had changed their way of thinking about their own movements and had been able to transfer the new knowledge to other everyday situations and with other people. They experienced having assimilated tools for changing their future movements, which they perceived as providing greater conditions for a sustainable everyday life. The students had also started to ask reflective questions in other learning situations with other people in their surroundings.

She didn't answer all the questions but just asked and then I had to think for myself … that was also a learning that I still do that I am … that I am self-critical … and if I have for example new students at work … I can't give the answer before I … that is I don't give the answer before I have put the question and allow the other to think for themselves. (student no. 10)

It has helped me to become aware of … yes about how I move and not just in terms of the movements in the study but yes in general. (student no. 5)

##### Experiencing cognitive impact for change

The students experienced that it is important for the changing process to think for themselves and “reformulate” the movement in the head, but thinking too much about one part of the movement appeared to disturb the whole performance.

When you are learning something new, you have to almost find others … perhaps not other muscles but perhaps use one's muscles in a different way or I don't quite know … but you have to almost think about how you want it … that is the body can't do it without you thinking in your brain how you want it and try to do it that way. (student no. 11)

An increased awareness of one's own movements and practice in everyday life situations was experienced as important prerequisites for changing movements. By watching one's own movement at home, the students can be supported in subsequently identifying which details need more practice. The films existed in the students’ heads for a long time thus gradually reducing the need for watching the recordings at home. The students also experienced that when the movements subsequently became more natural in their daily lives, they did not need to think as much about them.

I am sure that I think more often about bending my knees and keeping my arms close to my body when I lift heavy things. It feels more natural now, I don't need to think so much about it as I did before. (student no. 11, diary)

It's been like some sort of security having them there … but I haven't felt that I've needed to look at them because I've had them in my head all day (about the films). (student no. 7)

##### Experiencing change as meaningful

The students registered improvements in their own movements on the film, which was encouraging for them. They also discovered opportunities for improvements in their own posture and movement patterns, which were experienced as motivating for change.

Yes … then you become a bit more motivated so that it shows how you … you may perhaps not feel the same way yourself but you get an incentive by seeing that it's become better. (student no. 8)

The students experienced a reduced load on the body in everyday life when movement and work technique were adjusted, which for some students entailed a reduction in bodily fatigue at the end of the working day. The students put their own movements and posture patterns in relation to their discomfort, which increased their understanding and awareness of the relationship between pain and movement performance. To have the opportunity to influence one's own movement-related disorders both then as well as in the future was experienced as motivating. Prior to participating in the study, the students had expectations for increased movement awareness and improvements in their posture and movement pattern, which they associated with preventive measures for disorders in the future.

It feels better now … I didn't think that I did it in a certain way before starting with this but now when I compare I feel that it's easier and that it's more sparing for my body and that … it's easier to do it the way I'm doing it now that you … you get more power or so … it feels like that /… / when I've come home from work I've felt tired in my back and I don't think it's as much now … I think twice before doing anything when I'm going to lift something … or am going to do something that entails an effort. (student no. 11)

##### Being supported in self-reflection

The students experienced equality, interaction, and responsiveness in the conversation with the facilitator. To be able to initially describe one's own movements in words was experienced as being supportive for not only learning but also receiving guidance and, if necessary, a more hands-on tutorial. The students saw the facilitator's role as being supportive for their own reflection and the reflective questions as facilitating in detecting details and flaws in their own movement. The students also felt that the interviews provided support for reflection and helped to put the new knowledge into its context.

No but she (the facilitator) based her questions on my situation and always asked how I felt and I was to say what was good and less good before she said what she thought was good … she never criticized but this was for improving my way of walking or what should I call it /…/ it was a help I think … it was her way as a person I think. (student no. 10)

The students said that being reminded about how they move was important for the awareness and changes of movements to be sustained over time. Being able to watch one's own film and films of the role model at home was experienced as a security to come back to when needed. To have time for reflection and practice between the meetings was felt to be important for the long-lasting learning process.

#### Experiencing changes in one's own movement

To become aware of one's own posture and movement pattern through video feedback and reflection expanded the experience of the body and movements, and the video was considered to provide a clear image that cannot be distorted. The modified movements felt different in the body, and the inconvenient and unnatural feeling from the beginning changed during the process.

##### Experiencing a changed movement

The students were happy about the increased movement awareness in their everyday lives and said that this created a sense of security. The modified way of using the body may feel unnatural but gentler for the body at the same time. The students experienced that their everyday movements had become easier, more stable, and more controlled. In the beginning of the process, the changed movement may, however, feel unstable. For some students, the refined way of using the body was experienced as reducing the number of previous symptoms. The students felt that the body is used more and more as a whole, and that a small change in any part of the body changes the whole movement. A modified way of using the feet was emphasized as representing new knowledge that was sustained over time.

I have in a totally different way … mm … started to think about how I use my feet in the movements … feet are not just something you have socks and shoes on but are quite an important part in the actual movement and the movement pattern … and the feet still affect … I think now afterwards quite a lot how the body moves when walking … and I've started thinking about this a lot more and there aren't particularly big changes you need to do with your feet for the movement to be much better so I've started thinking about that much more. (student no. 6)

##### Changing the image of one's own movement

The students felt that they had assimilated valuable new knowledge about their own movements, which was an eye-opener. Watching oneself was experienced as providing greater knowledge of one's own posture and movement patterns, which was surprising and positive for the students. It provided a clear picture that prevented them from distorting their own internal image of the movement.

I think it was good because I got something to really think about … about how I work … I thought I had done right but I hadn't (laughter) … but it's not as though I think about it all the time but occasionally it comes up and I try to think about it but I believe that I … when I'm lifting something heavy and if I've got to pick up something from the floor then I try to lift with my whole body instead of just my arms. (student no. 8)

#### Experiencing challenges in the learning process

Long-lasting changes of posture and movement patterns were considered to be a challenge and a time-consuming process that needed reminders and feedback. A sense of uncertainty about getting it right can appear in everyday situations, and it can be frustrating to know how the movement should be and not be able to perform it in that way.

##### Experiencing uncertainty

The students experienced that it could be confusing to change a movement in the beginning. Feelings of uncertainty can appear when they are at home and are unsure whether they perform the movements correctly or not. The students felt that it could be difficult to detect the flaws in their own movements by themselves and could need help in doing that.

Yes … it's difficult to know how I do it myself … that is … sure I think about it and I think when I go up the stairs and how I did it in the study and how I should do it now … but it's difficult to know whether I'm doing it right because I can't see myself. (student no. 5)

##### Experiencing difficulties when changing

The students felt it to be frustrating to know how a movement should be performed when it did not turn out as desired. The students found it difficult at first to find the right movement and to describe with words how the movements were perceived. They could experience that habitual movement patterns felt good but in the films they did not look as the students wanted. They also found it difficult to change their habitual posture and movement patterns and that it took a long time to change. It was experienced as more difficult over time to remember to be attentive of one's own movements, especially in everyday life and in stressful situations. The parts of the movements they found complicated when in the room were also difficult to use afterwards, and some students felt that the selected movements were even difficult to use in everyday life.

It feels … a bit strange being as you're used to doing … if you've done something a hundred times and always done it that way then it's strange to change the pattern but you still notice that it's not as demanding when you do it that way. (student no. 9)

### Comprehensive understanding

Using an interactive learning model for movement awareness entails a powerful and challenging experience. It is experienced as meaningful and opens up for a reflective process within the individual. Ekebergh (2007) suggests that learning should start in a conscious reflection, which involves a person's whole lived experience and should be supported in an open and flexible way. The individual reflective process is continuous over time and leads to development of self-awareness. Inspired by Gadamer's theory of understanding development through the encounter between pre-understanding and new experiences (Gadamer, 1994), the model can be seen as a support for challenging the pre-understanding to extend the horizon of understanding, which can lead to a new understanding. The experience (in the situation) is a critical moment that remains in one's mind for a long time, which challenges the perception of one's own postural and movement patterns and experience of the bodily appearance. This implies new opportunities to access the surrounding world in a different way (Merleau-Ponty, 2002). Encountering the image of oneself also affects the way of thinking about one's own movements in relation to everyday situations, and the new knowledge thus becomes embodied and interwoven with everyday life experiences (Bengtsson, 2013; Merleau-Ponty, 2002). The feeling of usefulness gradually emerges and alternates with the feeling of discomfort when watching oneself on the recordings, and it is essential to have a trustful and safe atmosphere to allow this to occur. The model provides a space for testing and trying different kinds of performances in a permitting atmosphere, which positively contribute to the process of self-reflection and self-adjustment. Even though the movements that are drawn from everyday life are familiar, the awareness of the individual needs for development were clarified and embodied in the encounter with one's own movements.

## Discussion

In the study of nursing students’ experiences when using the interactive learning model, three themes and nine subthemes were formulated. The themes “Obtaining better preconditions for bodily awareness,” “Experiencing changes in one's own movement,” and “Experiencing challenges in the learning process” highlight the variety of experiences that include both difficulties and practical use. The use of the model was experienced as an eye-opener that initiated a process within the individual. Seeing and encountering one's own movements represents a critical moment that powerfully affects the individual and the reflection process continues in daily life situations and especially at work. An increased awareness of how the body is used in different everyday situations was developed, which might be considered to be a prerequisite for a long-lasting change. The experience of meaningfulness might contribute not only to the motivation for changing movement patterns in daily life over time to prevent MSS but also to change the bodily appearance. An increased awareness of one's own posture and movements provides opportunities to use the body in a different way that is smoother, gentler, and more effective. This could be seen as an increased access to the surrounding world, which we can experience through our bodies (Merleau-Ponty, 2002).

The video recordings may not always correspond to the perception of one's own movements or alignment but provide a complementary image of one's own movement that cannot be explained in the same manner as a performance in front of a mirror, etc. The video feedback affects experiences of and reflections regarding the bodily identity and contributes to the motivation for change. The concept of “embodied identity” has been described and studied by Gyllensten et al. (2010), describing that bodily awareness is inseparable from the identity and may affect the health of the individual.

Being video recorded and seeing oneself on the screen is not always a pleasant experience for the students. Some even expressed it as being unpleasant but at the same time they found the situation very useful and they were eager to learn more about their movement and alignment patterns. It is two-edged in the sense that feelings of usefulness emerge and alternate with feelings of discomfort. Based on the lifeworld perspective that views humans as lived subjects with lived experiences, it is possible to understand that a person can experience double feelings at the same time. This knowledge is important when implementing and using the model to prepare the learner for initial experiences of discomfort.

The body can be seen as an object, which at the same time is always a lived subject and everything we experience is understood through the subjective lived body (Merleau-Ponty, 2002). When watching one's own movement, the person in the video recording becomes an object in one sense while at the same time being a reflecting subject when watching the video. There is a continuously on-going dialogue between the learner and the facilitator supported by the video analysis tool. In the space that exists between the student and the facilitator in the encounter with the video recordings, something more is created (Merleau-Ponty, 2002). In this “between,” the facilitator learns how the student understands his/her own movement and what needs to be filled in or explained.

The atmosphere in the room was highlighted as being important for the students’ sense of safety and comfort in an already uncomfortable situation. The importance of trust and a permitting atmosphere is emphasized by Boud and Molloy (2013) as facilitating a well-functioning dialogic feedback. According to the authors, this is something often neglected in the higher education context. They further suggest that a useful strategy for achieving dialogic feedback is to involve students as assessors and to encourage the student to be the owner, the constructor, of the learning process (Boud and Molloy, 2013). This aspect is further described, but in a life-world perspective, by Ekebergh (2007), who suggests that all learning emanates from the lifeworld, that is, our lived experiences, pre-understanding, and approach. In the present learning model, this entails the learning starting in the experiences of the learner and he/she is the owner of the learning process. In this process, the facilitator's role and reflective and flexible approach are important. The results of this study show that being able to perform each movement several times and to test different ways help the student to feel more and more comfortable in the situation and contribute to a trustful and permitting atmosphere.

The learning model used in this study and the knowledge gained from the interviews with the students may be useful in different learning situations, when increased awareness of one's own movements and bodily appearance is desired. The development of individual awareness, supported by video-based feedback, is a powerful tool for making impressions last and for a long-lasting changing process. It may be an important addition to the planning of ergonomic education in, for example, nursing education, as well as in other practical learning contexts. This knowledge could also be of importance in the preventive work concerning MSS in general. Gyllensten et al. (2010) point out the importance of emphasizing problems in body awareness in patient treatment situations in, for example, the practice of physiotherapy. We suggest that this learning model might also be a valuable complement to movement awareness processes in various patient treatment situations.

In a recent systematic review and synthesis of interventions to reduce injuries in patient transfer situations, multicomponent interventions are strongly recommended and several components, for example, adequate training of all care staff, were highlighted as important (Thomas & Thomas, 2014). There is, however, a lack of detailed descriptions in current intervention studies about the content of the education and training and how it is performed. The learning model presented here might be a valuable complement for filling this gap of knowledge in terms of educational aspects of how learning in work technique and movement awareness can best be accomplished.

### Methodological considerations

The phenomenological hermeneutic approach chosen for the present study facilitated the understanding for and identification of the student's experiences of the learning model. The result is based on individual interviews on three different occasions, where the final one is a follow-up interview to gain a longer time perspective on the students’ experiences of the learning model when they had been working as a nurse. The students’ experiences varied to some extent over time, which is also described in the result, but as the aim of the study was to grasp experiences of the learning model, the data were analysed as a whole. The changes, however, raise more questions concerning the learning process over time, which could be a valuable focus in future studies.

There is never only one interpretation of a text (Ricoeur, 1976) and the result of this study represents one possible interpretation of the interviews and diary notes. Ricoeur and Thompson (1981) maintains that explanation entails bringing out the structure of a text and interpretation is to “follow the path of thought opened up by the text, to place oneself ‘en route’ towards the ‘orient’ of the text” (p. 162). He further sees interpretation as being a “particular province of understanding” (p. 150). The researchers have discussed different ways of understanding, interpreting, and explaining the data throughout the analysis process. This could be considered as strengthening the trustworthiness. In the natural attitude, judgements are made instantaneously, whereas in the analysis process we strove to put our judgements within brackets. This does not mean that we put our pre-understanding within brackets but we have, however, continuously asked each other if the description is based on what the text actually talks about to prevent our pre-understanding from steering the naïve reading, and in particular the structural analysis. The naïve understanding was rewritten after the first structural analysis being as the themes and subthemes were at first found to invalidate the naïve understanding. This process is in agreement with the description of the method. The comprehensive understanding is one possible interpretation of the structural analysis influenced by the naïve understanding as well as the chosen theoretical and philosophical perspectives (Lindseth & Norberg, 2004).

The number of students invited to participate was 105 and 11 took part in the whole study. This might be considered to be a low number of participants. The study was, however, an extracurricular activity that was performed during a period of clinical training. This might have affected the number of students who were able to participate. The authors’ intention had, however, been to include approximately 15–20 participants. All the participating students reported MSS during the past 12 months prior to the start of the study and several reported the effect on daily physical activities. This could possibly have affected the result of the study being as the students might have had a prior motivation to learn more about their movements and had expectations of getting help to reduce their symptoms.

## Conclusions

The present study provides greater knowledge about how a previously described interactive learning model for movement awareness is experienced by a group of nursing students. The model, which is based on a lifeworld perspective and contains video feedback, video modelling, reflection, diary writing, and videos for home practice, entails a powerful, challenging, and useful experience for developing movement awareness. Being aware of one's own movement affects the use and experience of the body in daily life, which in turn affects the access to the surrounding world. The experience of encountering oneself is etched in the mind of the student for a long time and motivates him/her to make changes in their daily life. It is experienced as useful for everyday life and as prevention for future symptoms. The feeling of usefulness gradually emerges and alternates with the feeling of discomfort when seeing oneself. In preventive interventions, such as ergonomic education, as well as in rehabilitation, learning aspects of movement awareness are essential but often overlooked. The presented learning model can be a useful contribution to this process.
